# Draft Genome Sequence of Escherichia coli DBS1, Isolated from a Patient with Urinary Tract Infections in Morocco

**DOI:** 10.1128/mra.00582-22

**Published:** 2023-02-23

**Authors:** H. Lemriss, S. Lemriss, A. Souiri, A. Hilali, S. El Kabbaj

**Affiliations:** a Laboratory of Technology and Health Sciences, Higher Institute of Health Sciences, Hassan 1st University, Settat, Morocco; b Department of Biosecurity PCL3, Laboratory of Research and Medical Analysis of Gendarmerie Royale, Rabat, Morocco; University of Maryland School of Medicine

## Abstract

Here, we present the draft genome assembly of Escherichia coli DBS1, which was originally isolated from a urine sample from a male patient with urinary tract infections in Rabat, Morocco.

## ANNOUNCEMENT

Urinary tract infections (UTIs) are among the most common bacterial infections and account for a significant part of the workload in clinical microbiology laboratories (1). Escherichia coli, are the most common etiological agents of UTIs, accounting for upwards of 75% to 90% of cases (2). Isolates from complicated uropathogenic E. coli (UPEC) infections are widely studied; however, there have been few reports characterizing uncomplicated UPEC infections, since urine culture is rarely included in routine diagnosis (3). In fact, UPEC infections present high genomic variability, making the identification of these pathogens a difficult task (4).

In this report, we present the draft genome sequence of Escherichia coli DBS1, isolated from a urine culture on UriSelect 4 chromogenic agar at 35°C for 48 h. The research described in this work was performed in adherence to the Declaration of Helsinki; no specific authorization was issued, since the samples were anonymized and no human data were used. DNA was extracted from a culture growing under the same conditions as above, using the PureLink genomic DNA kit (Invitrogen) according to the manufacturer’s recommendations.

The bacterium was identified using matrix-assisted laser desorption ionization–time of flight (MALDI-TOF) mass spectrometry (5) as Escherichia coli with a score value of 2.46, and the pure culture was stored at −80°C in 15% glycerol/5% tryptic soy broth.

A DNA library was prepared from 1 μg of genomic DNA, extracted following the Roche 454 Genome Sequencer (GS) rapid library preparation protocol. The genome sequence of Escherichia coli DBS1 was determined using high-throughput sequencing performed on a Genome Sequencer FLX+ system (454 Life Sciences, Roche) with FLX Titanium reagents, according to the manufacturer’s protocols. The 539,680,116 total reads (genome coverage, 44×; average length, 165 bp) produced using the GS FLX+ platform were quality controlled using Trimmomatic v0.36 (6), prior to assembly using Newbler v2.9 (7) with default parameters.

An automatic syntactic and functional annotation of the draft genome was performed using the MicroScope v3.11.1 platform (8, 9). The syntactic analysis combined a set of programs, including AMIGene v1 (10), tRNAscan-SE v1.21 (11), RNAmmer v1.2 (12), Rfam v13.0 scan (13), and Prodigal v2.6.3 software (14), to predict genomic objects that are mainly coding DNA sequences (CDSs) and RNA genes. A homology search was performed using UniProt (15) and more specialized databases, such as COG (16), InterPro v78.1 (17), PRIAM (May 2011; profiles for enzymatic classification) (18), TMHMM v2.0 (for prediction of protein localization) (19), SignalP v4.0 (20), and PSORTb v3.0.2 tools (21). Default parameters were maintained for all analyses.

The draft genome sequence comprises a total of 5,151,137 bp, consisting of 70 contigs (*N*_50_, 240,185 bp), with 50.40% G+C content, 5,100 genes, 4,912 CDSs, 43 pseudogenes, 3 rRNAs (5S,16S, 23S), and 77 tRNAs.

### Data availability.

The draft genome assembly for Escherichia coli DBS1 has been deposited at GenBank under the accession number FQSQ00000000.1. The version described in this paper is the first version. The draft genome raw data are available in the Sequence Read Archive (SRA) under accession number ERR6751196, BioProject accession number PRJEB15032, SRA accession number ERS1300622, and BioSample accession number SAMEA4389173.
